# Whole-Exome Sequencing and hiPSC Cardiomyocyte Models Identify *MYRIP*, *TRAPPC11*, and *SLC27A6* of Potential Importance to Left Ventricular Hypertrophy in an African Ancestry Population

**DOI:** 10.3389/fgene.2021.588452

**Published:** 2021-02-19

**Authors:** Marguerite R. Irvin, Praful Aggarwal, Steven A. Claas, Lisa de las Fuentes, Anh N. Do, C. Charles Gu, Andrea Matter, Benjamin S. Olson, Amit Patki, Karen Schwander, Joshua D. Smith, Vinodh Srinivasasainagendra, Hemant K. Tiwari, Amy J. Turner, Deborah A. Nickerson, Dabeeru C. Rao, Ulrich Broeckel, Donna K. Arnett

**Affiliations:** ^1^Department of Epidemiology, University of Alabama at Birmingham, Birmingham, AL, United States; ^2^Department of Pediatrics, Children’s Research Institute, Genomic Sciences and Precision Medicine Center, Medical College of Wisconsin, Milwaukee, WI, United States; ^3^College of Public Health, University of Kentucky, Lexington, KY, United States; ^4^Cardiovascular Division, Department of Medicine and Division of Biostatistics, Washington University, St. Louis, MO, United States; ^5^Division of Biostatistics, Washington University, St. Louis, MO, United States; ^6^Department of Biostatistics, University of Alabama at Birmingham, Birmingham, AL, United States; ^7^Department of Genome Sciences, University of Washington, Seattle, WA, United States

**Keywords:** exome sequencing, left ventricular hypertrophy, echocardiograph, cardiomyocyte model, hypertension, African-American

## Abstract

**Background**: Indices of left ventricular (LV) structure and geometry represent useful intermediate phenotypes related to LV hypertrophy (LVH), a predictor of cardiovascular (CV) disease (CVD) outcomes.

**Methods and Results:** We conducted an exome-wide association study of LV mass (LVM) adjusted to height^2.7^, LV internal diastolic dimension (LVIDD), and relative wall thickness (RWT) among 1,364 participants of African ancestry (AAs) in the Hypertension Genetic Epidemiology Network (HyperGEN). Both single-variant and gene-based sequence kernel association tests were performed to examine whether common and rare coding variants contribute to variation in echocardiographic traits in AAs. We then used a data-driven procedure to prioritize and select genes for functional validation using a human induced pluripotent stem cell cardiomyocyte (hiPSC-CM) model. Three genes [myosin VIIA and Rab interacting protein (*MYRIP*), trafficking protein particle complex 11 (*TRAPPC11*), and solute carrier family 27 member 6 (*SLC27A6*)] were prioritized based on statistical significance, variant functional annotations, gene expression in the hiPSC-CM model, and prior biological evidence and were subsequently knocked down in the hiPSC-CM model. Expression profiling of hypertrophic gene markers in the knockdowns suggested a decrease in hypertrophic expression profiles. *MYRIP* knockdowns showed a significant decrease in atrial natriuretic factor (*NPPA*) and brain natriuretic peptide (*NPPB*) expression. Knockdowns of the heart long chain fatty acid (FA) transporter *SLC27A6* resulted in downregulated caveolin 3 (*CAV3*) expression, which has been linked to hypertrophic phenotypes in animal models. Finally, *TRAPPC11* knockdown was linked to deficient calcium handling.

**Conclusions**: The three genes are biologically plausible candidates that provide new insight to hypertrophic pathways.

## Introduction

Changes in left ventricular (LV) structure and geometry may evolve with myocardial injury or pressure overload and can lead to chamber dilation and/or hypertrophy (Gaasch and Zile, 2011). Echocardiography can identify such changes, while providing useful intermediate phenotypes predictive of cardiovascular (CV) disease (CVD) outcomes (Vasan et al., 2009). Left ventricular mass (LVM), used clinically to define left ventricular hypertrophy (LVH), is an independent predictor of premature mortality across all genders, races, and ages (Casale et al., 1986; Levy et al., 1990; Gaasch and Zile, 2011). Additionally, increased LV wall thickness predicts CVD events (Vasan et al., 1997b), and LV dilation predicts heart failure (Vasan et al., 1997a; Gardin et al., 2006).

It is widely accepted that genetic factors influence LVM and other structural cardiac phenotypes. Heritability estimates for LVM (Sharma et al., 2006), LV internal diastolic dimension (LVIDD), and LV relative wall thickness (RWT) range between 25 and 50% (Fox et al., 2010; Jin et al., 2011). Linkage (Arnett et al., 2009a; Fox et al., 2010), candidate gene association (Arnett et al., 2009a; Lynch et al., 2012), and genome-wide association studies (Arnett et al., 2009b, 2011; Vasan et al., 2009) have reported loci suggestively or significantly associated with structural and functional echocardiographic traits.

Similar to the increased risk of hypertension and other CVDs, individuals of African ancestry (hereafter AAs) have greater average LV structural dimensions and other LV structural differences relative to individuals of European ancestry. LVH contributes more to the risk of CV mortality in AAs than it does in other ancestry groups (Havranek et al., 2008). Also, AAs have a higher prevalence of concentric LVH characterized by an increased LVM and RWT (hypertrophic LV growth without overall chamber enlargement), which has a poorer prognosis and is usually the result of increased systolic pressure (Gaasch and Zile, 2011; Ahmad et al., 2017). Overall, the extent to which genetic factors influence differences in LV phenotypes and the unique etiology of subclinical LVH in hypertension-prone AAs remain poorly understood.

We conducted a whole-exome association study of structural echocardiographic traits, including LVM adjusted to height^2.7^ (LVM/Ht^2.7^), LVIDD, and RWT among AAs from the Hypertension Genetic Epidemiology Network (HyperGEN; Williams et al., 2000). Both single-variant and gene-based association tests were performed to examine the association between common and rare coding variants and echocardiographic traits in AAs. We sought functional validation of the association findings using a human induced pluripotent stem cell cardiomyocyte (hiPSC-CM) model.

## Materials and Methods

### Study Population

Hypertension Genetic Epidemiology Network is one of the four networks in the National Heart, Lung and Blood Institute’s Family Blood Pressure Program, which aimed to identify genetic contributors to hypertension. As part of HyperGEN, hypertensive AA sibships were recruited from the community-at-large in Birmingham, AL, and population-based cohorts in Forsyth County, NC, from 1995 to 2000. Sibling pairs with onset of hypertension before age 60 years were recruited in the first phase. In a second phase, the study was extended to include other siblings as well as offspring of the hypertensive probands who were not being treated for hypertension. This study included 1,434 self-reported AA participants with relevant echocardiographic measurements and exome sequence data. The study was approved by the Institutional Review Boards of the participating organizations. All HyperGEN participants provided informed consent for use of samples and data for subsequent analyses.

### Echocardiographic Data

Left ventricular traits were assessed by two-dimensional-guided M-mode echocardiography at the Birmingham, AL, and Forsyth County, NC field centers following a standardized imaging and reading protocol. All instruments were calibrated against a standard phantom at installation and were validated regularly (Tang et al., 2002). Certificated sonographers from each center were trained at the echocardiography reading center at New York Hospital-Weill Cornell Medical Center where measurements were computerized, calibrated, and quantified using a review station with digitizing tablet and monitor overlay (Arnett et al., 2009a).

The study assessed LV structural phenotypes including LVM, LVIDD, and RWT. LVM was calculated with the following formula: LVM = 0.8 × 1.04 × [(IVS + LVIDD + PWT)^3^ − LVIDD^3^] + 0.6 g, in which IVS is the interventricular septum thickness and PWT is the posterior wall thickness at end-diastole (in cm). IVS, PWT, and LVIDD were measured according to the American Society of Echocardiography recommendations (Devereux et al., 1984a). LVM was further indexed to height in meters^2.7^. RWT was calculated as twice the PWT divided by the LVIDD (Devereux et al., 1984b).

### Exome Sequence Data

Exome sequencing was performed at the University of Washington (Auer et al., 2015). The processes of library construction, exome capture, sequencing, and mapping were performed as previously described (Tennessen et al., 2012). Samples of DNA were quality controlled by concentration estimation by PicoGreen® (Thermo Fisher Scientific, Waltham, MA, United States). DNA samples were prepared by subjecting genomic DNA to shearing followed by ligation of sequencing adaptors. Exome capture for the samples was performed using the Roche Nimblegen SeqCap® EZ (Roche, Pleasanton, CA, United States) according the manufacturer’s instructions. Paired-end sequencing (2 × 76 bp) was performed using GAII® and HiSeq® (Illumina, San Diego, CA, United States) sequencing instruments. For quality control (QC) purposes prior to release of sequence data, samples were initially converted from real-time base-calls to qseq.txt files using Illumina’s standard base caller, Bustard, and aligned to hg19 human reference using Burrows-Wheeler Aligner (Li and Durbin, 2010). Duplicate removal and insertion-deletion (INDEL) realignment were performed using the Genome Analysis ToolKit (GATK®, Broad Institute, Cambridge, MA; McKenna et al., 2010). After using GATK filters, samples were required to reach at least 20x coverage over 70% of the exome target. Prior to release of individual-level sequence reads, sequence data were required to match known fingerprint genotypes for their respective samples.

### Variant Calling Approach

All subjects were whole-exome sequenced at average mean read depth was 25. GATK’s best practice for calling germline variants is a multistep approach, which invokes the following GATK modules: (a) HaplotypeCaller module to generate per-sample, intermediate genomic variant call format (GVCF) files; (b) ImportGenomicsDB module to consolidate sample-level GVCF files; (c) GenotypeGVCF module to jointly call single-nucleotide polymorphisms (SNPs) and INDELs; and (d) VariantRecalibrator and ApplyRecalibration modules to retain high-quality variants and calibrate them to balance sensitivity and specificity. This multi-step approach produced a total of 642,878 variants across the 1,434 samples, which were further reduced to 528,450 variants with Variant Quality Score Log-Odds scores >0.99 derived from GATK’s Variant Quality Score Recalibration.

### Sample Quality Control

To ensure retention of high-quality samples relevant to our research study, 1,434 genomic samples were reduced to 1,382 through a quality-assurance pipeline that involved (a) removal of duplicate or twin sib pairs; (b) removal of blood sample mix-ups; and (c) removal of one non-AA sample through estimation of genetic principal components (PCs).

Additional quality assessment metrics were evaluated to retain high confidence samples and variants for downstream statistical analysis. After aligning the generated paired-end read sequences, the following QC steps were implemented: (a) total reads: exome completion typically requires a minimum of 30 M PE50 reads; (b) library complexity: the ratio of unique reads to total reads mapped to target (DNA libraries exhibiting low complexity are not cost-effective to finish); (c) capture efficiency: the ratio of reads mapped to human vs. the reads mapped to target; (d) coverage distribution: 90% at ≥8x required for completion; (e) capture uniformity; (f) raw error rates; (g) Ti/Tv ratio: typically 3.2 for known sites and 2.9 for novel sites (Wang et al., 2015); (h) distribution of known and novel variants relative to dbSNP (Ng et al., 2009); (i) fingerprint concordance >99%; (j) homozygosity; (k) heterozygosity; and (l) sample contamination validation. All QC metrics for both single-lane and merged data were reviewed by a sequencing analyst to identify data deviations from known or historical norms.

### Variant Quality Control

To minimize false positives, only the following variants were retained: (a) genotyping quality > 20; (b) depth of coverage > 8; (c) bi-allelic variants (i.e., SNPs only); and (d) sample-level genotype missing rate of < 10%. We included only autosomal SNPs. The above SNP-filtering criteria reduced the SNP space from 528,450 to 397,723, with 353,686 SNPs having a minor allele frequency (MAF) < 5%, of which 134,548 (38%) SNPs were singletons. The working set of 397,723 SNPs was further annotated using ANNOVAR and variants with MAF < 5% were classified into categories of replacement mutations [i.e., missense (nonsynonymous) and loss-of-function (stop-gain and stop-loss) mutations, see Supplementary Table S1]. Based on these annotations, a total of 202,697 were deemed probable deleterious and damaging variants: 199,104 missense mutations; 3,454 stop-gain; and 139 stop-loss. ANNOVAR was used to capture functional prediction scores and classifications.

### Statistical Analysis

A total 1,364 participants of 1,382 had genetic and echocardiographic data available for this analysis. Prior to performing genetic association tests, echocardiographic traits were transformed as needed to meet distributional assumptions (natural log transform of LVM/Ht^2.7^, LVIDD, and RWT). To address potential population stratification, we performed PC analysis using EIGENSTRAT’s Smartpca module with variants with MAF > 5%. The first 10 PCs were found to capture ancestral variation and hence were used as covariates in the downstream statistical analysis.

Genetic association was assessed at both the single-variant level [with minor allele count (MAC) > 5] as well as the gene level (no MAC filter) using RAREMETAL and RAREMETALWORKER (RMW; Liu et al., 2014), which reports results from gene-based tests including the Sequence Kernel Association Test (SKAT-RMW) which adjusts for family relationship. Since RMW does not include a function for joint testing of rare and common variants and our hypothesis centered around this combination of variants, we additionally implemented SKAT (Wu et al., 2011) outside of the RMW framework, with a focus on the rare/common SKAT function (SKAT-RC; Ionita-Laza et al., 2013). Models were adjusted for age, sex, recruitment center, and the top 10 PCs as fixed effects and kinship coefficients for sample relatedness within families (SKAT-RMW output only). In a sensitivity model, we additionally adjusted for body mass index [BMI, weight (kg)/height^2^ (m^2^)] and in a second sensitivity analysis we adjusted for hypertension status. The Bonferroni-corrected significance thresholds for single variant and gene-level tests were *p* < 2.5 × 10^−7^ (i.e., 0.05/202,697 variants) and *p* < 3.01 × 10^−6^ (i.e., 0.05/16,579 genes), respectively. However, we used a gene-based test *p* < 1 × 10^−4^ and other information to prioritize genes for cell model experiments (see Gene Selection section below).

### hiPSC-CM Models

#### hiPSC-CM Cell Lines and Hypertrophy Model

We utilized iCell® Cardiomyocytes (iCell-CMs) from hiPSCs [Cellular Dynamics International (CDI), Madison, WI, United States] to determine mRNA expression patterns; this particular line was derived from the fibroblast of a Caucasian female (<18 years old) using the retroviral transduction reprogramming method (Cellular Dynamics, 2020). These cardiomyocytes are a highly pure ventricular population having functional properties similar to adult human cardiomyocytes (Kattman et al., 2011; Ma et al., 2011). iCell-CMs were plated at 2.0 × 10^4^ cells/well in a 96-well plate pre-coated with fibronectin (5 μg/ml). After 10 days of recovery in iCell Maintenance Medium (CDI), the cells were cultured in William’s E medium supplemented with Cocktail B (1:25) from the Hepatocyte Maintenance Supplement Pack (Life Technologies Gibco®, Thermo Fisher Scientific) for an additional 4 days.

After a total of 14 days of culture, cells were harvested with Total RNA Purification 96-Well Kit (Norgen Biotek Corp, Thorold, ON, Canada). Total RNA was extracted per manufacturer’s recommendations, resuspended in nuclease-free water, and quantified by UV spectrophotometry (NanoDrop 2000, Thermo Fisher Scientific). Quality of total RNA was evaluated using total RNA Pico chip analysis on Agilent 2,100 Bioanalyzer (Agilent Technologies, Santa Clara, CA, United States).

#### Illumina Hiseq Sequencing

We analyzed gene expression for the gene prioritization and selection analysis using RNA sequencing (RNA-seq). Paired-end cDNA libraries were prepared from 500 ng of total RNA from the iCell cardiomyocytes, using the TruSeq® RNA Sample Preparation Kit (Illumina). External RNA Controls Consortium (Baker et al., 2005) reference materials were added to each RNA sample per manufacturer’s recommendations. Samples were sequenced on the Illumina HiSeq instrument following the manufacturer’s instructions. The samples were sequenced to a depth of at least 40 million paired-end reads per sample.

#### Gene Selection for hiPSC-CM Knockdown Experiments

Since all gene-based statistical tests include assumptions that may not perfectly fit the underlying genetic model, we prioritized genes for functional follow-up with a combination of criteria using a point-counting approach similar to that of previous work in HyperGEN (Zhi et al., 2012). We required genes to be: (1) associated with at least one structural trait (LVIDD, RWT or LVM/Ht^2.7^ with *p* < 1.0 × 10^−4^); (2) previously associated broadly with cardiovascular disease or metabolism in the literature; and (3) expressed in the hiPSC-CM model (see Illumina HISeq Sequencing section above) for a total of three points. The following four criteria were supportive: (1) association (*p* < 0.05) with more than one of the structural traits, (2) harboring a loss of function variant, (3) previously linked to hypertrophy in the literature, and/or (4) having a mix of rare and common variants contributing to the gene-based statistic. To prioritize genes for functional validation analysis using knockdown experiments, we summed the count of each criteria for a total of seven possible points and prioritized the gene selection by the total count. This procedure resulted in four genes with a prioritization score of 5. One of the four genes, prostaglandin E receptor 3 (*PTGER3*), was not practical for knockdown in our model due to its role in cell signaling (i.e., PTGER3 is a prostaglandin receptor and cardiomyocytes do not produce prostaglandins).

#### Knockdown Experiments

Gene knockdown experiments were conducted in iCell-CMs using commercially available small interfering (si) RNAs (Thermo Fisher Scientific) targeting the identified candidate genes. hiPSC-CMs were plated in 96-well dishes at an approximate density of 1.71 × 10^4^ cells/well. Transfections were performed in triplicate using Invitrogen’s Lipofectamine® RNAiMAX reagent (Thermo Fisher Scientific) per manufacturer’s instructions. Each siRNA transfection was conducted using two unique Ambion® siRNAs (Thermo Fisher Scientific) per gene to ensure efficient gene knockdown. Invitrogen’s Block-iT™ fluorescent control (Thermo Fisher Scientific) was used as a positive control, with successful nuclear-localized fluorescence. Two negative controls were run with no discernable effect: (1) the transfection reagent without siRNAs and (2) Ambion® Silencer™ Negative Control No. 3 siRNA (Thermo Fisher Scientific). RNA extractions were performed with Norgen’s Total RNA purification 96-well kit (Norgen Biotek Corp). The knockdown efficiency was determined *via* real-time quantitative PCR (RT-qPCR) using TaqMan™ (Roche) gene expression assays following the manufacturer’s recommendations, normalizing to GAPDH, and compared to the Silencer Negative Control No. 3.

#### Targeted Whole-Transcriptome Sequencing

Targeted whole-transcriptome RNA sequencing was performed using the Ion AmpliSeq™ Transcriptome Human Gene Expression Kit (Thermo Fisher Scientific) on total RNA extracted from the iCell-CM knockdown experiments. Barcoded cDNA libraries were generated from 10 ng of total RNA, following manufacturer’s instructions. Quality of libraries was confirmed and quantification was done using Agilent Bioanalyzer High Sensitivity DNA Kit (Agilent Technologies). Sequencing was performed on the Ion Torrent™ Ion Proton™ sequencing platform (Thermo Fisher Scientific), using the Ion PI Hi-Q Sequencing 200 Kit. Three biological replicates were run for each condition.

#### Hypertrophic Gene Markers in siRNA Knockdown Experiments

Genes previously linked to hypertrophy, as reflected by an increase in LV wall thickness and/or LV dimensions, were selected to test for a hypertrophic expression profile in the knockdown cells (Carlson et al., 2013). Differential gene expression analysis in knockdown experiments was performed using the R/Bioconductor package DESeq2 (Love et al., 2014). Only genes with median depth of ≥10 reads were considered. In particular, genes relevant to muscle contraction and regulation were evaluated, including actin genes [actin alpha cardiac muscle 1 (*ACTC1*), actin alpha 1, skeletal muscle (*ACTA1*), actinin alpha 1 (*ACTN1*)]; mammalian heart myosin heavy chain genes [myosin heavy chain 6 (*MYH6*), myosin heavy chain 7 (*MYH7*)]; and the actin-binding protein gene transgelin (*TAGLN*; Warkman et al., 2012; Despond and Dawson, 2018). A number of other markers were also evaluated. Natriuretic peptide A (*NPPA*) and natriuretic peptide B (*NPPB*) are evolutionarily conserved genes expressed by the heart during the embryonic and fetal stages. In adulthood, upon cardiac stress, *NPPA* and *NPPB* are upregulated in the ventricular myocardium (Man et al., 2018). Corin, serine peptidase (*CORIN*) is a cardiac serine protease that acts as a pro-atrial natriuretic peptide convertase (Wang et al., 2008). F-box protein 32 (encoded by *FBXO32*, aliases *MAFbx1*, and *ATROGIN1*) is a cardiac and muscle specific F-box protein with E3 ligase activity that localizes at the sarcomere (Al-Yacoub et al., 2016). Tripartite motif containing 63 (*TRIM63*) encodes muscle RING finger 1, which maintains muscle protein homeostasis by tagging the sarcomere proteins with ubiquitin for subsequent degradation by the ubiquitin-proteasome system and imparts loss-of-function effects on E3 ligase activity, which is causal for hypertrophy (Chen et al., 2012). Programmed cell death 4 (*PDCD4*) is upregulated during cardiomyocyte apoptosis and is involved in the inflammatory response and cellular differentiation (Jia et al., 2016).

#### Ingenuity Pathway Analysis

We used Ingenuity Pathway Analysis (IPA, QIAGEN, Inc., https://www.ingenuity.com) software to analyze the differential mRNA expression gathered from the candidate gene knockdowns. Pathway analysis uses observed expression changes and combines these findings with predicted gene regulation and expression changes in associated network genes and gives different information than that provided by the knockdown models. Due to our focus on echocardiographic traits, we selected cardiovascular pathways to analyze in depth. We examined IPA’s canonical cardiovascular signaling pathways including cardiac hypertrophy signaling and cardiac beta-adrenergic signaling. Additionally, we analyzed the pathway “Aldosterone signaling in epithelial cells.” Aldosterone has been recognized as being potentially hypertrophic (Yamamuro et al., 2006; Messaoudi et al., 2013). We also analyzed the canonical toxicity pathways for mitochondrial function and NRF2-mediated stress responses. Finally, we looked at metabolism changes since a switch from fatty acid (FA) metabolism to glucose metabolism in cardiomyocytes can be indicative of cardiomyopathy (Pascual and Coleman, 2016). This included IPA canonical pathways for FA beta-oxidation and *PPARA*/*RXRA* activation. Pathways could have either positive or negative *z*-scores for activation or repression of pathways, respectively. The reported *z*-score helps to infer the activation or repression of the implicated biological functions based on the relationship between the observed molecular network patterns and the derived effect based on the literature.

## Results

### Study Population

Characteristics of the HyperGEN population are described in Table 1, which compares participants above and below the median value of LVM/Ht^2.7^ (note we dichotomized the phenotype for this table only; discovery genetic models were of continuous LV traits). On average, those with above-median LVM/Ht^2.7^ were older, more likely male, had higher BMI, and were more likely to be diabetic. This group also had higher values of other structural indices (RWT and LVIDD).

**Table 1:** Descriptive characteristics of the Hypertension Genetic Epidemiology Network (HyperGEN) African ancestry (AA) cohort.

	N (%) or Mean (SD)	Below Median LVM/Ht^2^	Above Median LVM/Ht^2^	*p*
*N*		682	682	
Age (years)	49.1 (11.2)	48.0 (11.3)	50.2 (11.1)	<0.001
Female (%)	877 (64.3)	512 (75.1)	365 (53.5)	<0.0001
Field site				0.037
Alabama (%)	1,010 (74.0)	517 (75.8)	493 (72.3)	
North Carolina (%)	315 (23.1)	140 (20.5)	175 (25.7)	
Missing site data (%)	39 (2.9)	25 (3.7)	14 (2.0)	
BMI (kg/m^2^)	32.0 (7.5)	29.9 (6.4)	34.1 (7.9)	<0.0001
Diabetes (%)	294 (21.6)	120 (17.6)	174 (25.5)	<0.001
LVM/Ht^2.7^ (g/m^2.7^)	43.6 (12.6)	35.2 (7.0)	51.9 (11.4)	<0.0001
RWT	0.34 (0.05)	0.33 (0.05)	0.35 (0.06)	<0.0001
LVIDD (cm)	5.2 (0.5)	4.8 (0.3)	5.5 (0.5)	<0.0001

BMI, indicates body mass index; LVIDD, left ventricular internal dimension at diastole; LVM/Ht^2.7^, left ventricular mass adjusted to height in meters^2.7^; and RWT, relative wall thickness.

### Single Variant Tests

There was no single variant with MAC > 5 that met the genome-wide significance level threshold after correction for multiple testing (*p* < 2.5 × 10^−7^) for the three echocardiographic traits. Supplementary Figure S1 shows the QQ plots for single variant analysis with (a) no variant filter, (b) MAC > 1 filter, and (c) MAC > 5 filter. Three variants with MAC > 5 in three different genes [regenerating family member 3 gamma (*REG3G*) for lnRWT, activating transcription factor 7 interacting protein 2 (*ATF7IP2*) for lnLVIDD, and A-kinase anchoring protein 12 (*AKAP12*) for lnLVIDD] approached statistical significance (Supplementary Table S2). As the majority of the borderline significant SNP findings were very rare (MAC < 5), gene-based results became the focus of the analysis and were used to select genes to carry forward to functional cell-model assays.

### Gene-Based Tests and Gene Prioritization

Table 2 includes the gene-based results limited to the three genes with the highest gene prioritization score (=5) selected for knockdown in the hiPSC-CM model [myosin VIIA and Rab interacting protein (*MYRIP*), trafficking protein particle complex 11 (*TRAPPC11*), and solute carrier family 27 member 6 (*SLC27A6*)]. Each gene was associated with a structural trait (lnLVIDD), expressed in the hiPSC-CM model, and had SKAT-RC *p* < 1 × 10^−4^ for a total of three votes. Adjusting for BMI and, separately, hypertension in the gene-based association test models did not substantially change the results for the three genes. Each gene received two additional votes corresponding to supportive prioritization criteria. Single variant statistics for markers contributing to gene-based tests, gene expression data from the hiPSC-CM model, and fulfillment of gene prioritization categories are presented in the Supplementary Spreadsheet. For *MYRIP*, the two additional gene prioritization criteria were fulfilled by the contribution of both rare and common variants to the gene-based statistic (22 rare and two common) and that one of those variants was a rare loss of function variant (3:40085643, MAF = 0.0004). The average MAF of the 22 rare variants was 0.002 and the two common variants (3:40223771 and 3:40275461) had MAF 0.23 and 0.10, respectively. *SLC27A6* had 13 variants (average rare variant MAF 0.006) contributing to the gene-based statistics, with one variant being common (5:128301885, MAF = 0.21) fulfilling one additional prioritization criteria. The other criterion was met by an existing publication reporting a common (MAF = 0.37) 5’ UTR variant associated with LVH (Auinger et al., 2012). In *TRAPC11* both rare and common variants as well as a loss of function variant were observed fulfilling two prioritization criteria. The 22 rare variants had average MAF = 0.005, including the loss of function variant (4:184633795) with MAF = 0.0004.

**Table 2:** Genes selected for functional follow up in human induced pluripotent stem cell cardiomyocyte (hiPSC-CM) models by prioritization scheme^¶^.

Trait	Gene	Total Vars	Rare	Avg MAF	SKAT-RC^*^ *p*	SKAT-RC^§^ *p*	SKAT-RC^%^ *p*	SKAT-RMW^†^ *p*
lnLVIDD	*SLC27A6*	13	10	0.006	3.37 × 10^−6^	2.58 × 10^−7^	8.10 × 10^−6^	3.38 × 10^−6^
lnLVIDD	*TRAPPC11*	22	17	0.005	4.71 × 10^−5^	4.15 × 10^−4^	4.07 × 10^−5^	2.91 × 10^−4^
lnLVIDD	*MYRIP*	24	21	0.002	3.93 × 10^−5^	2.86 × 10^−5^	8.11 × 10^−6^	5.14 × 10^−3^

Avg MAF indicates average minor allele frequency; LVIDD, left-ventricular internal diastolic dimension; SKAT-RC, sequence kernel test-rare/common function; SKAT-RMW, sequence kernel test-rare metal worker function; and Vars, variants.

* Adjusted for age, sex, recruitment center, and 10 ancestry principal components (Pcs).

§ Additionally adjusted for body mass index.

% Additionally adjusted for hypertension status.

† Corrected for family relationships.

¶ Each gene was required to be associated with a structural trait [LVIDD, RWT, or left ventricular mass (LVM) adjusted to height in meters^2.7^] with *p* < 1 × 10^−4^, expressed in the hiPSC-CM model, and broadly related to cardiovascular disease in the literature for a total of three points, additional points were accumulated for (1) Having a loss of function variant [myosin VIIA and Rab interacting protein (*MYRIP*), trafficking protein particle complex 11 (*TRAPPC11*)], (2) Reference related to hypertrophy solute carrier family 27 member 6 (*SLC27A6*), (3) Having a mix of rare and common variants contribute to the gene-based statistic (*MYRIP*, *SLC27A6*, and *TRAPPC11*), or (4) Associated with more than one echo trait with *p* < 0.05 (not observed for top genes) for a possible total of seven points.

### Candidate Gene hiPSC-CM Knockdowns

Each of the three prioritized genes was successfully knocked down in the cell model using gene-specific siRNA. We used RT-qPCR to show the efficiency of the knockdown procedures (Supplementary Figure S2) before performing target whole transcriptome sequencing. *SLC27A6* proved the most efficient knockdown at 96.5%, while *TRAPPC11* and *MYRIP* each showed significant expression reduction at 85.4 and 80.3%, respectively, compared to control cells transfected with Silencer Negative Control #3.

### Cardiac Hypertrophy Markers

**Figure 1:**
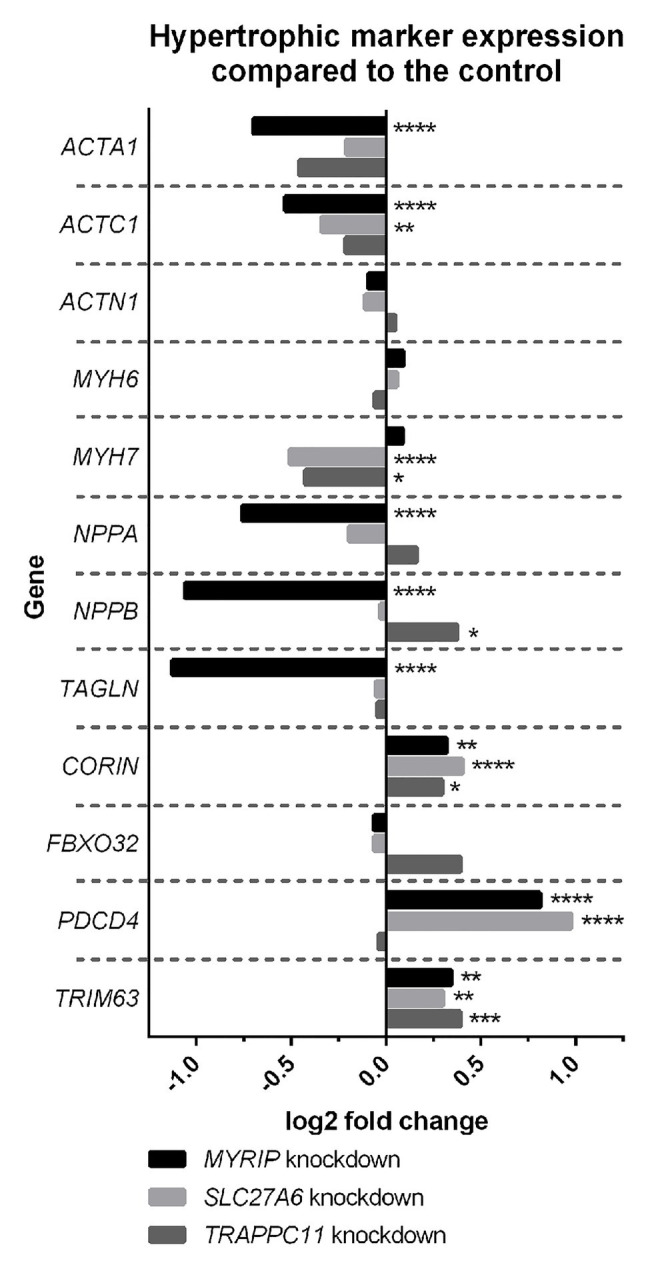
Hypertrophic marker expression compared to control. Some hypertrophic genes experienced significant changes in expression in the knockdown model. False discovery rate (FDR), value of ^*^p < 0.05, ^**^p < 0.01, ^***^p < 0.001, and ^****^p < 0.0001.

Expression profiling of hypertrophic gene markers in the hiPSC-CM knockdowns overall suggest a decrease in hypertrophic expression profiles (Figure 1). *SLC27A6* and *MYRIP* knockdowns both showed significant decrease in the expression of actin genes *ACTA1* and *ACTC1*. Knockdown of *SLC27A6* and *TRAPPC11* showed significant decreases in *MYH7*. *MYRIP* knockdowns showed a very significant decrease in *NPPA* and *NPPB* expression. *TRAPPC11* knockdowns, however, showed a significant increase in *NPPB* expression. *PDCD4* was highly upregulated in both *SLC27A6* and *MYRIP* knockdowns.

### Pathway Analysis of MYRIP Knockdowns

**Figure 2:**
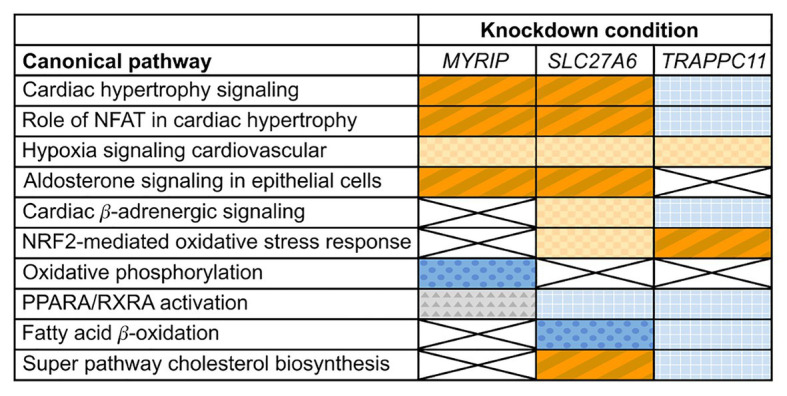
Changes in expression profiles of pathways relevant to cardiac hypertrophy in candidate gene knockdowns. Orange (diagonal stripes, checkerboard), blue (dots, grid), and gray (triangles) colored boxes indicate significant changes in experimental pathway expression compared to the control (*p* ≤ 0.05). Dark blue (dots) indicates a significant negative *z*-score (≤−1) and a deactivation of the pathway, while dark orange (diagonal stripes) indicates a significant positive *z*-score (≥+1) and activation. Light colored (checkerboard, grid) and gray boxes indicate insignificant *z*-scores. White boxes marked with an X had insignificant pathway expression changes (*p* ≥ 0.05).

Differential expression analysis of *MYRIP* knockdowns compared to the control identified changes in expression of cardiac hypertrophy pathways. Both “Cardiac hypertrophy signaling” and the “Role of NFAT (nuclear factor activated T cells) in cardiac hypertrophy” pathways had significantly positive *z*-scores greater than +1 (+2.4 and +3.1, respectively) suggesting pathway activation (Figure 2) characterized by increased expression of receptors, intermediate signalers, and downstream hypertrophic effectors. The aldosterone-signaling pathway for the *MYRIP* knockdown model was also scored as significantly upregulated with a positive *z*-score of +2.3. Within this model (Supplementary Figure S3), there is a significant increase in expression of the aldosterone mineral corticoid receptor nuclear receptor subfamily 3 group C member 2 (*NR3C2*). Furthermore, within this pathway there is a significant increase in the expression of protein kinase C epsilon (*PRKCE*) and the Na^+^/H^+^ antiporter *SLC9A1* when compared to control. Differential expression pathway analysis showed a significant decrease (*z*-score −5.2) in oxidative phosphorylation in *MYRIP* knockdowns. Over 70% of genes involved in the canonical oxidative phosphorylation pathway experienced a significant decrease in expression compared to the control, while only 10% of genes were upregulated.

### Pathway Analysis of SLC27A6 Knockdowns

**Figure 3:**
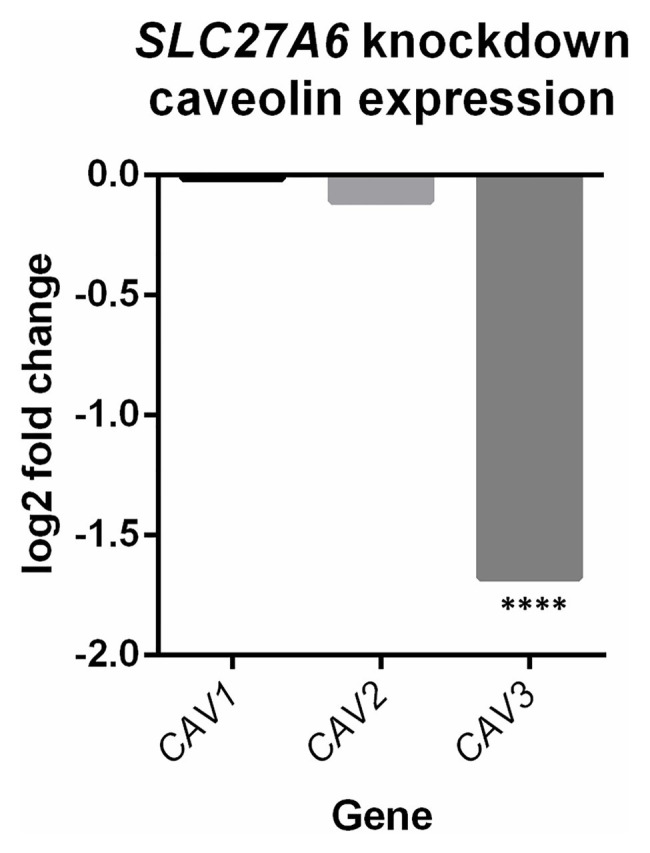
*SLC27A6* knockdowns show a decreased expression of caveolin 3 (*CAV3*) compared to the control. A significant decrease in *CAV3* expression was noted in the *SLC27A6* knockdowns. ^****^FDR *p* < 0.0001.

Differential expression of *SLC27A6* knockdowns predicted significant upregulation of the “Cardiac hypertrophy signaling” and “Role of NFAT in cardiac hypertrophy” pathways (*z*-scores +1.9 and +1.7, respectively; see Figure 2). Aldosterone signaling was also predicted to be upregulated (*z*-score +2.3) due to the significantly increased expression of PKC genes [like *PRKCE* and protein kinase D3 (*PRKD3*)] and mitogen activated protein kinase phosphatase 1 (*MPK1*). Of particular interest to the *SLC27A6* knockdowns was the significant upregulation among all genes associated to cholesterol biosynthesis compared to the control. *CAV3*, which codes for the caveolin protein in myocytes, was significantly downregulated in the IPA pathway (Supplementary Figure S4) and consistently we observed significantly decreased expression of *CAV3* in the *SLC27A6* knockdown cardiomyocytes (value of *p* 1.64 × 10^−5^), supporting a hypothesis of disrupted caveolae formation (Figure 3).

### Pathway Analysis of TRAPPC11 Knockdowns

Comparison of *TRAPPC11* knockdowns to the control showed insignificant expression changes in canonical hypertrophic pathways (Figure 2). Cardiac β-adrenergic signaling was predicted to be downregulated with significant decrease in the expression of Na^+^/Ca^2+^ exchanger member *SLC8A1* and the ryanodine receptor 2 (*RYR2*). Such a predicted decrease could lead to a deficiency in Ca^2+^ handling (Supplementary Figure S5). Furthermore, *TRAPPC11* knockdowns showed more definitive pathway changes for peroxisome proliferator-activated receptor alpha/retinoid X receptor alpha (*PPARA*/*RXRA*) activation compared to *MYRIP* and *SLC27A6*. *RXRA* and some other downstream effectors had elevated expression leading to an overall predicted upregulation of this pathway.

## Discussion

Increased left ventricular dimensions are prognostic of a host of cardiovascular outcomes that overburden individuals of African Ancestry. Among AAs, hypertension is associated with abnormalities in left ventricular structure, including both concentric (increased LVM with increased RWT and normal or decreased LVIDD) and eccentric (increased LVM with increased LVIDD) forms of hypertrophy (Abdalla et al., 2016). Heritability studies (Sharma et al., 2006; Fox et al., 2010; Jin et al., 2011) as well as prior genome-wide association investigations (Arnett et al., 2009b, 2011; Vasan et al., 2009) suggest genes contribute to variability in these phenotypes. Previous studies have mostly focused on common genetic variation and identified markers that explain only a small portion of echocardiographic trait variation. We undertook a whole-exome sequencing analysis of structural and functional echocardiographic traits in the AA participants of the HyperGEN study and used a data-driven gene prioritization procedure to select genes for validation in a hiPSC-CM model. Three genes were prioritized and successfully knocked down in the cells. Compared to control cells, knockdown cells showed altered expression of genes in cardiovascular pathways providing new insight into disease-relevant mechanisms.

MYRIP is a Rab effector protein, which facilitates melanosome transport and is detected in multiple tissues including the heart. It is also is involved in secretory vesicle transport, such as from the adrenal chromaffin cells (where catecholamines such as epinephrine, norepinephrine, and dopamine are produced and released systemically; Desnos et al., 2003). In our study, *MYRIP* knockdown was associated with significantly decreased expression of *NPPA* and *NPPB*, 2 adjacent and highly conserved genes that have a complex relationship to hypertrophic phenotypes. In our cell models, a decreased expression appeared to be protective for LVH. However, in the context of cardiac stress (e.g., cardiac ischemia), NPPA and NPPB expression increases in response to pressure overload-induced hypertrophy in a compensatory response (Man et al., 2018). An inadequate response by these proteins may favor natriuresis and ventricular dilation (i.e., increased LVIDD; Tamura et al., 2000; Man et al., 2018). *MYRIP* also significantly changed expression of actins (*ACTA1* and *ACTC1*) and *TAGLN*, myocyte structural proteins. Variants in *ACTC1* have been associated with idiopathic dilated cardiomyopathy and familial hypertrophic cardiomyopathy (Augiere et al., 2015; Frustaci et al., 2018). Differential expression analysis of *MYRIP* knockdowns compared to the control in IPA also showed changes in aldosterone signaling that included an increase in expression of the aldosterone mineral corticoid receptor, *NR3C2*. The mineral corticoid receptor is widely expressed in the cardiovascular system and is a major determinant of endothelial function, smooth muscle tone, vascular remodeling, and hypertension (Belden et al., 2017). Individuals of AA are particularly prone to hypertension associated with primary aldosteronism (Zilbermint et al., 2019). A potential upregulation of this pathway further validates a role of *MYRIP* in left ventricular remodeling. Finally, based on gene expression, there was a very significant predicted decrease in the regulation of the oxidative phosphorylation pathway in *MYRIP* knockdowns, suggesting a state of relative energy deprivation characteristic of LVH and heart failure (Scolletta and Biagioli, 2010).

*SLC27A6* encodes a member of the FA transport protein family (FATP6), the predominant FA transporter in the heart. In the healthy heart, ATP requirements are generally met by FA oxidation with smaller contributions from other sources (such as glucose, lactate, and branch-chain amino acids); increased reliance on non-FA fuels is characteristic of a “fetal program” of substrate metabolism typical of LVH (Neubauer, 2007; Tuunanen et al., 2008). *SLC27A6* has also been linked to other CV-related outcomes in humans (Nielsen et al., 2018; Roselli et al., 2018; Van Der Harst and Verweij, 2018). With the knockdown of *SLC27A6*, IPA also predicted decreased expression of caveolin 3 (*CAV3*). This relationship was confirmed in our cell model. Caveolae are cholesterol- and sphingolipid-enriched membrane microdomains that contain caveolins, structural proteins that are involved in cell growth and hypertrophy by organizing receptors and signaling molecules (including G-protein coupled receptors and natriuretic peptide receptors; Horikawa et al., 2011). There are three caveolin isoforms, and previous research has shown *Cav3* knockout mice show signs of muscle myopathic changes, cardiac hypertrophy, and cardiomyopathy (Woodman et al., 2002). Upregulation of *Cav3* in a neonatal cardiac myocyte model reduced the ability of an adrenergic agonist (i.e., phenylephrine) and endothelin-1 to provoke hypertrophy in that model (Koga et al., 2003). Another study showed that transgenic mice with cardiac myocyte-specific overexpression of *Cav3* that were subjected to transverse aortic constriction had increased survival, reduced cardiac hypertrophy, and maintenance of cardiac function compared with control mice. Mutations in *Cav3* have also been linked to the long QT type 9 inherited arrhythmia syndrome (Tyan et al., 2019). As predicted by IPA as a consequence of *SLC27A6* downregulation was *SLC27A1* (alias *FATP1*) upregulation, which may be compensatory. In the heart, overexpression of *SLC27A1* leads to LVH and LV diastolic dysfunction similar to that seen with diabetic cardiomyopathy (a consequence of high circulating FAs and the insulin-resistant heart unable to bring in glucose; Chiu et al., 2005; Wu et al., 2006). Overall, a downregulation of *SLC27A6* may lead to downregulation of *CAV3* and upregulation of *SLC27A1*, which is plausibly involved in ventricular remodeling. *PDCD4*, which was upregulated in the *SLC27A6* knockdown, may be another signal of increased cardiac apoptosis that is associated with cardiac stress, resulting in an increase in LVIDD (i.e., eccentric LVH; Xiao et al., 2016).

Transport protein particles (TRAPP) are parts of a multiprotein complex involved in endoplasmic reticulum-to-Golgi trafficking (Sacher et al., 1998; Kim et al., 2006; Scrivens et al., 2011). It is ubiquitously expressed in humans, reflecting its important role in basic cellular functions. The TRAPPC11 protein participates in numerous interactions with other TRAPP complex components. Similar to *SLC27A6*, *TRAPC11* knockdown was associated with fewer alterations in hypertrophic marker expression as compared to *MYRIP*. In IPA, a decrease in cardiac β-adrenergic signaling was linked to a decrease in the expression of the Na^+^/Ca^2+^ exchanger and *RYR2*, potentially leading to a deficiency in Ca^2+^ handling. Overall, a downregulation *TRAPPC11* supports a defect in the transport of secretory proteins, potentially involving Ca^2+^ that warrants further investigation.

This study leverages several strengths, including a whole-exome association discovery analysis of echocardiographic traits in a population of well-characterized participants of AA. We used a combination of statistical analysis, publicly available bioinformatics data, and gene expression data from our control cell models to prioritize our top findings. This resulted in a novel list of genes for functional follow-up in hiPSC-CMs. Limitations included the inability to test specific variants or groups of variants identified in human exomes in the cell model, where instead we used whole-gene siRNA knockdown as a proxy for gene disruption due to identified variants. Additionally, human induced pluripotent stem cell derived cardiomyocytes are known to not fully replicate the maturity of human cardiac myocytes. However, in this study, we used the commercially available iCell cardiomyocytes for the knockdown experiments, which were developed to represent a highly pure population of cardiomyocytes. Prior research performed a detailed quantitative analysis of the electrophysiological properties of these cells demonstrating several properties characteristic of mature cardiac myocytes (Ma et al., 2011). Further, the genes were not replicated in an independent population due to the difficulty finding comparable whole exome data paired with echocardiography in AAs. Also, these findings represent genes discovered in a relatively healthy population and validated in a model of normal cardiomyocytes. Future studies are needed to determine a role for these genes in diseased individuals and cell models (or even additional *in vivo* models).

The current study discovered three novel candidates (*MYRIP*, *SLC27A6*, and *TRAPPC11*) associated with LV internal diastolic dimension in AAs from HyperGEN. The three genes were successfully knocked down in a hiPSC-CM model and markers of hypertrophy were compared between the knockdown and controls cells. Knockdown of each gene was associated with significant cellular changes that give new insight into left-ventricular remodeling. In particular, *MYRIP* was associated with cardiac hypertrophy signaling (e.g., NFAT pathway, NPPA, and NPPB) and aldosterone signaling. *SLC27A6* is a fatty acid transporter and our knockdown of the gene led to changes in caveolin expression (*CAV3*) whose protein product has previously been linked to hypertrophic phenotypes in animal models. Finally, *TRAPPC11* was linked to a deficiency in calcium handling and *RYR2* expression. In summary, the three genes are biologically plausible candidates associated with left ventricular structure and metabolism according to the results of the knockdown experiments. Future functional studies that focus on specific variants under normal and stress conditions in the hiPSC-CM models are warranted.

## Data Availability Statement

The authors acknowledge that the data presented in this study must be deposited and made publicly available in an acceptable repository, prior to publication. Frontiers cannot accept a manuscript that does not adhere to our open data policies.

## Ethics Statement

The studies involving human participants were reviewed and approved by University of Kentucky Institutional Review Board University of Alabama at Birmingham Institutional Review Board Washington University in Saint Louis Institutional Review Board Medical College of Wisconsin Institutional Review Board University of Washington Institutional Review Board. The patients/participants provided their written informed consent to participate in this study.

## Author Contributions

MI, DR, UB, and DA contributed to the conception and design of the study. MI, PA, SC, BO, and AT drafted sections of the manuscript. MI, CG, HT, and DR guided the statistical analyses. AD, AP, KS, and VS managed data and conducted the statistical analyses. JS and DN oversaw and conducted whole-exome sequencing. AM, BO, AT, and UB oversaw and conducted hiPSC laboratory experiments and associated analyses. LF provided expertise on the phenotypes and interpretation of the biological significance of findings. All authors contributed to manuscript revision, read, and approved the submitted version.

### Conflict of Interest

The authors declare that the research was conducted in the absence of any commercial or financial relationships that could be construed as a potential conflict of interest.The handling editor declared a past co-authorship with the authors CG, MI, KS, and DR.

## Figures and Tables

**Table:** 

Term	Definition
AA	African ancestry
AAs	participants of African ancestry
BMI	body mass index
CV	cardiovascular
CVD	cardiovascular disease
FA	fatty acid
GATK®	Genome Analysis ToolKit
GVCF	genomic variant call format
hiPSC-CM	human induced pluripotent stem cell cardiomyocyte
HyperGEN	the Hypertension Genetic Epidemiology Network (study)
iCell-CMs	iCell® Cardiomyocytes
INDEL	insertion-deletion genetic variant
IPA	Ingenuity Pathway Analysis
IVS	interventricular septum thickness
LV	left ventricular
LVH	left ventricular hypertrophy
LVIDD	left ventricular internal diastolic dimension
LVM	left ventricular mass
LVM/Ht2.7	left ventricular mass adjusted to height2.7
MAC	minor allele count,
MAF	minor allele frequency
NFAT	nuclear factor activated T cells
PC	principal component
PWT	posterior wall thickness at end-diastole
QC	quality control
RMW	RAREMETALWORKER (analysis tool)
RT-qPCR	real-time quantitative polymerase chain reaction
RWT	relative wall thickness
siRNA	small interfering RNA
SKAT-RC	rare/common function in sequence kernel asssociation test
SKAT	Sequence Kernel Asssociation Test (analysis tool)
SNP	single-nucleotide polymorphism
