# Supplementary material for: Whole-Exome Sequencing and hiPSC Cardiomyocyte Models Identify MYRIP, TRAPPC11, and SLC27A6 of Potential Importance to Left Ventricular Hypertrophy in an African Ancestry Population
Source: Front Genet. 2021 Feb 19;12:588452. doi: 10.3389/fgene.2021.588452 (PMC7933688; doi:10.3389/fgene.2021.588452)
Supplement: Supplementary file 1 [file Data_Sheet_1.pdf]

Supplementary Material

1 Supplementary Figures and Tables

1.1 Supplementary Figures

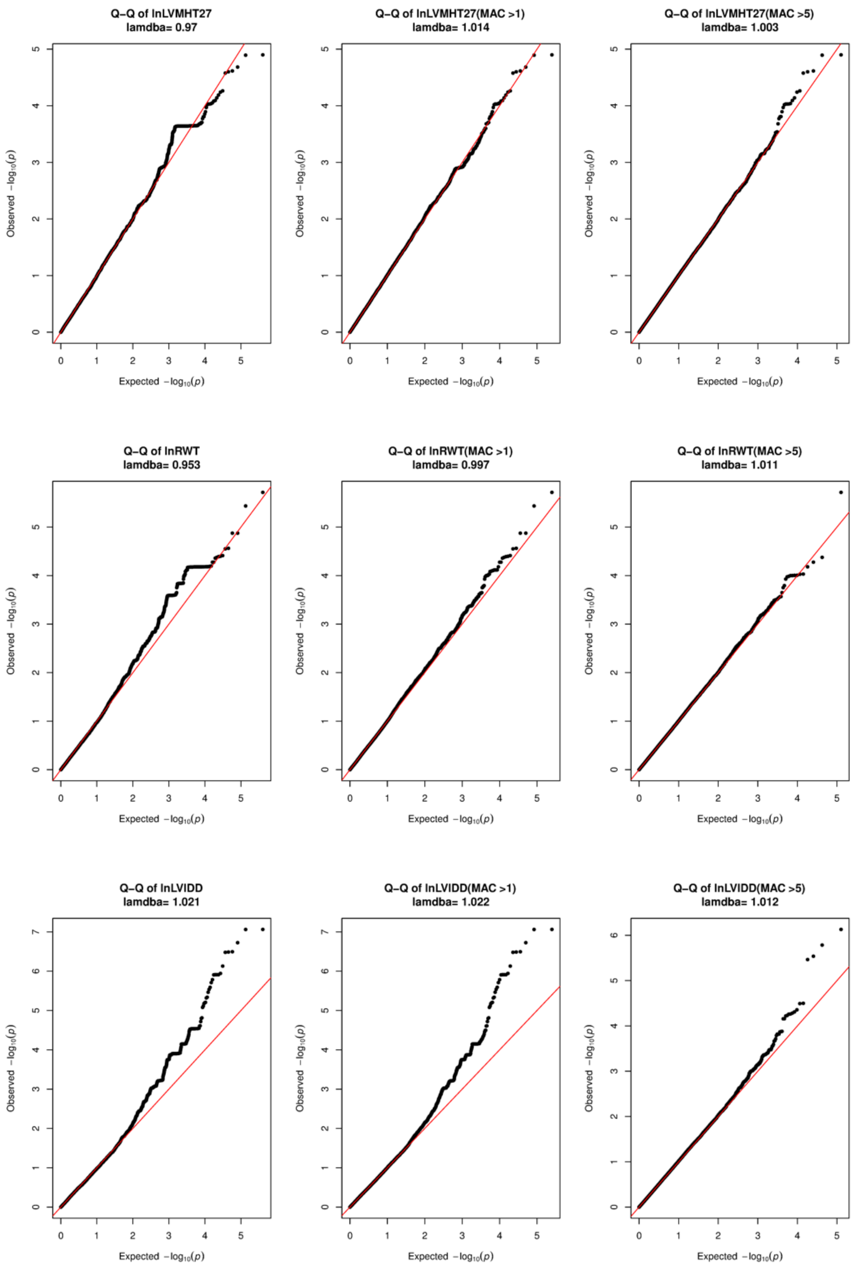

Supplementary Figure 1. Single variant QQ plots of nonsynonymous variants for 3 echocardiography traits including three plots for each trait all variants (left column), MAC>1 (middle column), MAC>5 (right column). LVMHT27 indicates left-ventricular mass (g) indexed to height$^{2.7}$ (m$^{2.7}$); MAC, minor allele count; RWT, relative wall thickness.

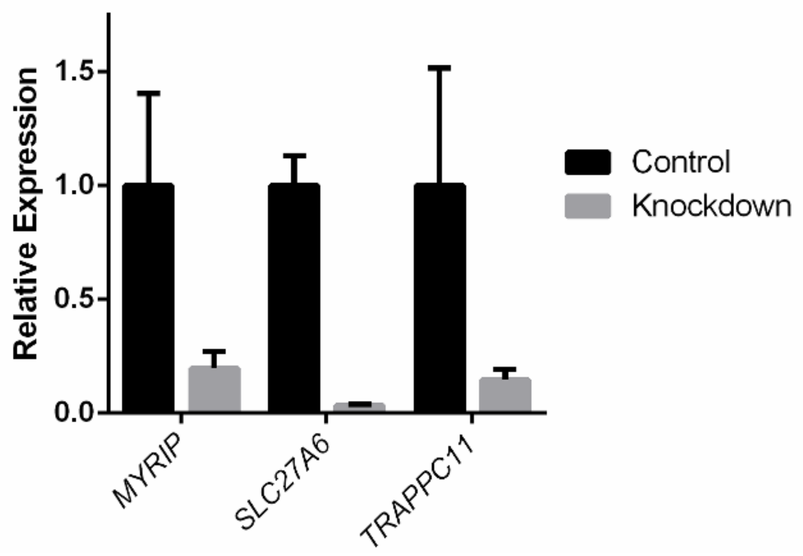

Supplementary Figure 2. Prioritized genes were successfully knocked down by siRNA. Knockdown expression is relative to the candidate gene expression in the control. Graph includes data from both biological replicate rounds of knockdown. Error bars represent standard deviation. Solute carrier family 27 member 6 (*SLC27A6*) experienced 96.5% reduction (*P*=2.04×10-17); trafficking protein particle complex 11 (*TRAPPC11*) experienced 85.4% reduction (*P*=1.70×10-5); myosin VIIA and Rab interacting protein (*MYRIP*) experienced 80.3% reduction (*P*=8.84×10-6).

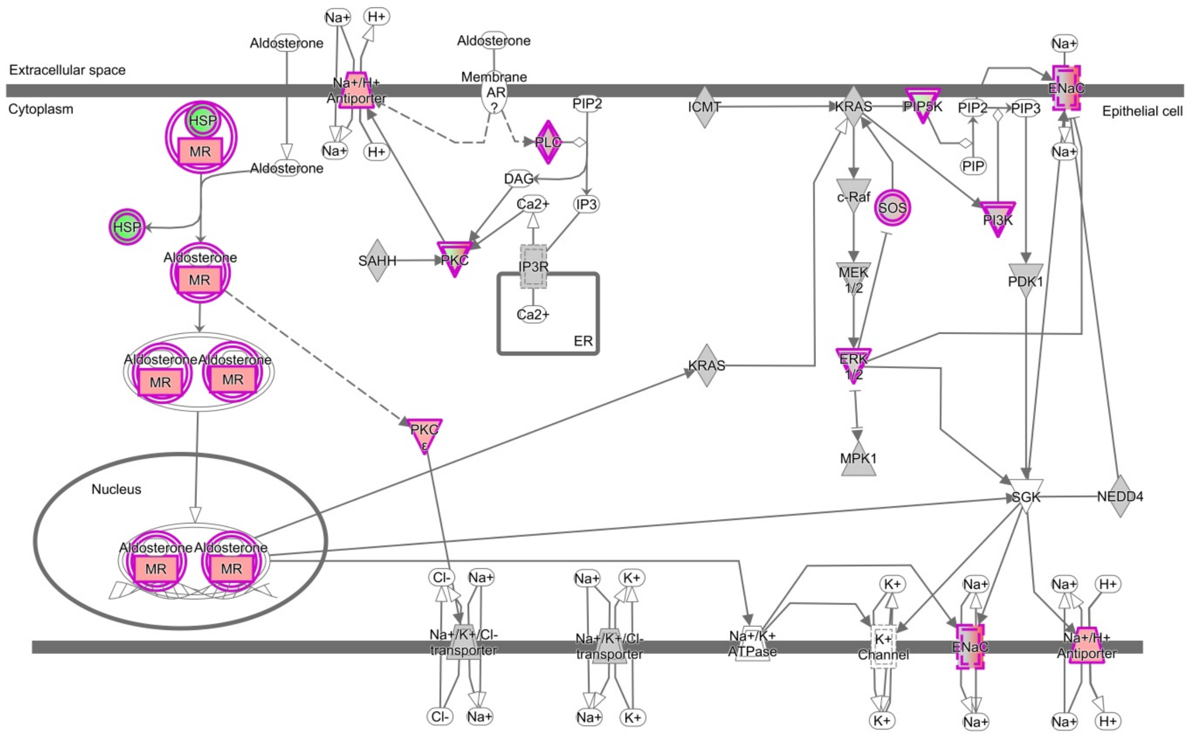

© 2000-2019 QIAGEN. All rights reserved.

Supplementary Figure 3. Ingenuity pathway analysis showing increase in expression of the aldosterone mineral corticoid receptor (*NR3C2*), protein kinase C (*PKC*) and the Na+/H+ antiporter with myosin VIIA and Rab interacting protein (*MYRIP*) knockdown.

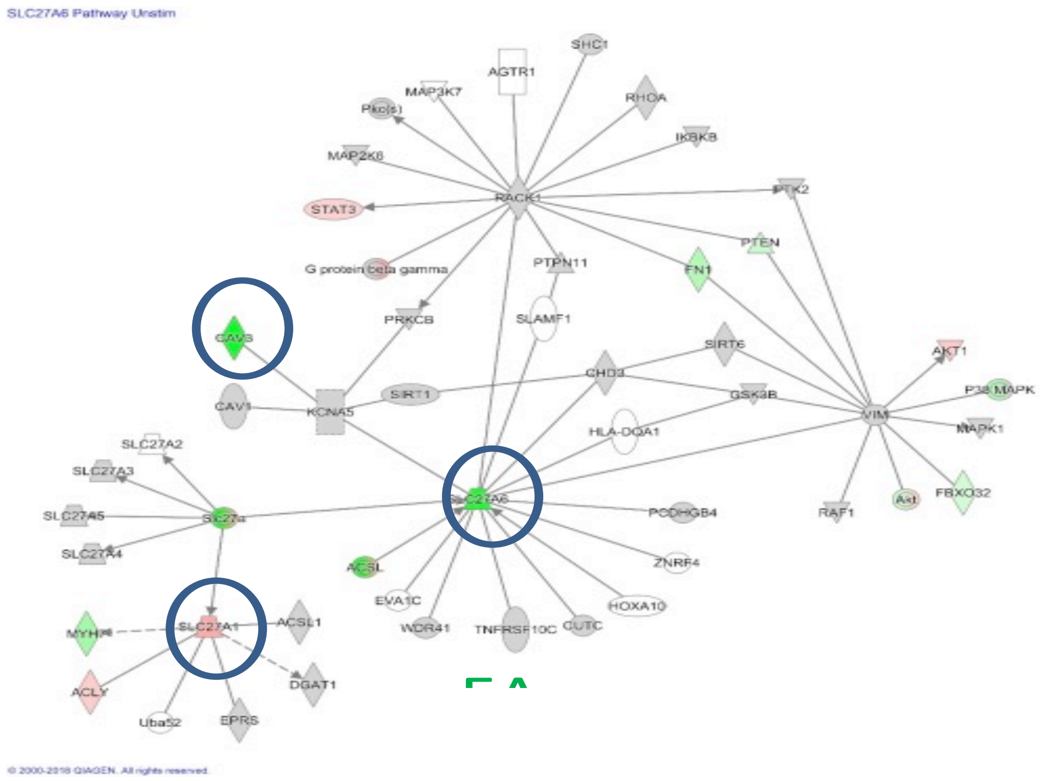

Supplementary Figure 4. Ingenuity Pathway Analysis showing solute carrier family 27 member 6 (*SLC27A6*) with decreased expression (green) as well as caveolin 3 (*CAV3*) with decreased expression (green). *SLC27A1* shows increased expression (red).

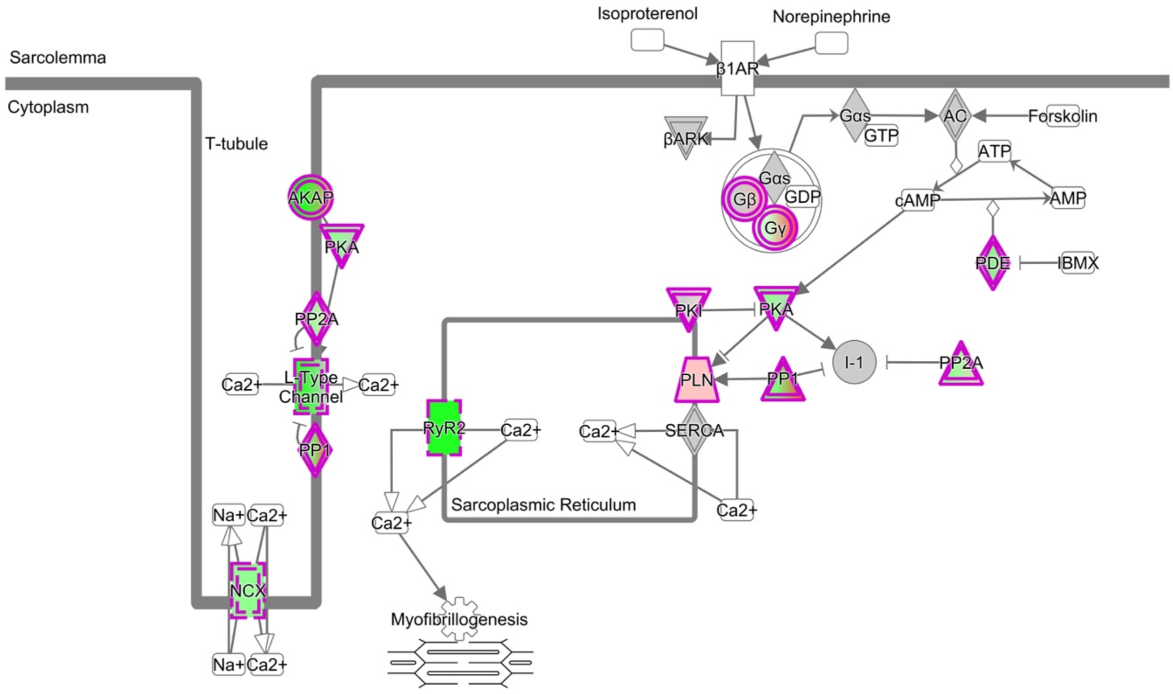

© 2000-2019 QIAGEN. All rights reserved.

Supplementary Figure 5. Ingenuity Pathway Analysis suggests that cardiac β-adrenergic signaling is downregulated with major decrease in the expression Na^{+}/Ca^{+2} exchange and ryanodine receptor 2 (*RYR2)* (green).

1.2 Supplementary Tables

Supplementary Table 1. Functional classification of called variants

Func.refGene	ExonicFunc.refGene	Variant Count
downstream	-	252
exonic	nonsynonymous	199104*
exonic	stopgain	3454*
exonic	stoploss	139*
exonic	synonymous	149001
exonic	unknown	3258
exonic;splicing	nonsynonymous	46
exonic;splicing	synonymous	22
exonic;splicing	unknown	1
intergenic	-	5303
intronic	-	25771
ncRNA_exonic	-	2267
ncRNA_exonic;splicing	-	6
ncRNA_intronic	-	1417
ncRNA_splicing	-	10
splicing	-	1516
upstream	-	368
upstream;downstream	-	101
UTR3	-	2464
UTR5	-	3211
UTR5;UTR3	-	12

*included in single variant and gene-based analysis

Supplementary Table 2. Top results for single variant analysis

Trait	CHR	POS	REF	ALT	N	MAF	Beta	SD	PVALUE	Genomic Region	Gene	SNP Function
lnRWT	2	79254949	G	A	1365	0.06	-0.059	0.012	1.87E-06	Exonic	*REG3G*	nonsynonymous
lnLVIDD	16	10524821	C	T	1365	0.005	0.128	0.052	7.43E-07	Exonic	*ATF7IP2*	nonsynonymous
lnLVIDD	6	151670689	C	T	1365	0.002	0.251	0.025	1.69E-06	Exonic	*AKAP12*	nonsynonymous

MAF indicates minor allele frequency; Beta indicates beta coefficient; SD standard deviation; CHR, chromosome; LVIDD, left ventricular internal diastolic dimension; REF indicates reference allele; ALT indicates alternate allele; POS, position; RWT, relative wall thickness
